# The comprehensive characterization of *Prosopis juliflora* pods as a potential bioenergy feedstock

**DOI:** 10.1038/s41598-022-22482-9

**Published:** 2022-11-03

**Authors:** G. Gayathri, Kiran Babu Uppuluri

**Affiliations:** Bioprospecting Laboratory, Centre for Bioenergy, School of Chemical and Biotechnology, SASTRA Deemed University, Thanjavur, 613 401 India

**Keywords:** Environmental sciences, Environmental chemistry, Environmental biotechnology, Renewable energy

## Abstract

The production of renewable and sustainable biofuels using inevitable wastes is a promising alternative to the alarming depletion of fossil fuels. Significantly, the sustainable biorefinery of lignocellulosic waste, as an alternative fuel source, is a prognosticating approach to tackle many agricultural/forestry residues and offers a circular economy as well as environmental benefits. But, the heterogeneity of lignocellulosic biomass is one of the major bottlenecks in lignocellulosic biorefinery. Thus the characterization of lignocellulosic biomass is essential to understanding the feedstock's nature, composition and suitability for biofuel production. The present study taps evergreen spiny non-edible pods of *Prosopis juliflora* (Pj) as an energy feedstock. Proximate, ultimate and biochemical characterization of Pj pods were conducted, and thermal behaviour and calorific values were determined. Cellulose and hemicellulose were isolated and characterized by reliable methods. The overall characterization has revealed the Pj pods as a potential feedstock for bioenergy. The collected Pj pods contain (% w/w) moisture 7.89 ± 0.002, volatile matter 87.67 ± 0.002, ash 0.21 ± 0.002, fixed carbon 4.23 ± 0.002 with a calorific value of 17.62 kg/MJ. The CHNS content was (w/w %) carbon 41.77, nitrogen 3.58, sulfur 26.3 and hydrogen 6.55. The biochemical composition analysis yields (% w/w) on a dry basis; cellulose 26.6 ± 0.18, hemicellulose 30.86 ± 0.27, lignin 4.71 ± 0.12, protein 11.63 ± 0.12 and starch 1.1 ± 0.06 and extractives 30.56 ± 0.008. The isolated cellulose and hemicellulose were analyzed and confirmed by CP/MAS &^1^H NMR, FTIR, TG-DSC, SEM, XRD, and TGA. The present results revealed that the tested biomass, *Prosopis juliflora*, could be used as a feedstock in biorefinery for bioenergy.

## Introduction

The foundation of modern civilization, energy is essential to most human activities. Modernizing our lives and cities would not have been possible without energy^1^. Energy demand is anticipated to increase by around 50% over the next 20 years, with predictions of 778 EJ by 2035^2^. But the exhaustion of fossil fuels and the subsequent increase in greenhouse gas emissions have become urgent problems^3^. These harmful greenhouse gases also harm human health because they cause cancer^4^. Thus finding an alternative energy source that can satisfy energy needs also be environmentally benign is vital^4^. In contrast to other environmentally friendly energy sources, including wind, solar, geothermal, marine, and hydropower, biomass may directly produce fuel and chemicals^5^.

Lignocellulosic biomass comes in various wastes, including woody, aquatic detritus, farm manure and byproducts, and agricultural wastes^6^. The energy from the lignocellulosic biomass is greatly interesting since it is abundantly available, technically feasible and economically viable^7^. Several technologies, including gasification, combustion, pyrolysis, enzymatic hydrolysis and fermentation, are employed to turn biomass into fuel and platform chemicals^3^. Thermochemical processes often demand a large amount of energy, including a solvent or catalyst. In contrast, the biochemical approach has a protracted time and has less effective at breaking down the resistant biomass^3^. Various lignocellulosic biomass like sugarcane bagasse^8^, berry waste^2^, jatropha seeds^9^, sunflower straw^10^, sorghum stem^11^, rice straw^12^, waste peach pulp^13^, wheat straw^14^, corn stover^15^ and so forth have been studied for the effective production of biofuels.

The lignocellulosic feedstock contains majorly cellulose 40–50%, hemicellulose 25–35% and lignin 15–20%^16^. Cellulose is the primary polymer that contains long chains of cellobiose units, a renewable resource that is safe for the environment, inexpensive, non-toxic, biodegradable, and biocompatible^3,17^. Hemicellulose comprises monosaccharides such as mannose, galactose, arabinose, glucose, rhamnose, xylose and uranic acid^18^. Lignin is a high molecular weight, complex structure containing cross-linked phenolic monomers that provide cell wall rigidity, impermeability and resistance against microbial attack^19^.

The lignocellulosic biomass usage in biorefineries for biofuels offers reduced greenhouse gas emissions, zero waste generation and significant sustainability in the circular bioeconomy^4^. Nevertheless, there is a constant demand for environment-friendly disposal or refinery of various lignocellulosic biomass^20^. From the standpoint of waste valorization, the fractionation of this lignocellulosic residue into cellulose, hemicellulose, and lignin is the primary step for producing a variety of products with a higher economic value, such as biofuel, cosmetics, biodegradable films, paper, and plastic materials, while minimising the associated environmental impacts^19^.

One of the most promising technologies for converting lignocellulosic biomass into high-value products like biochar, syngas, and bio-oil without oxygen is pyrolysis^21^. By recycling trash using pyrolysis to create biochar, energy, and value-added goods, agricultural and animal waste disposal can lessen its negative environmental effects^22^. Biochar production from lignocellulose is simple, reliable, technically feasible and commercially viable^23^. Using biochar as a feed for animal growth reduces methane formation and enhances anaerobic digestion^23^.

The present study aims to exploit one such lignocellulosic biomass, *Prosopis juliflora pods,* for its suitability as bioenergy feedstock. The *Prosopis juliflora* (Pj) plant is a drought-tolerant, evergreen tree with drooping branches of the *Leguminosae* family as an energy stock^24^. The native of the Pj pods to arid and semi-arid regions of the world. Pj plants have been colonized in many countries, such as North America, Africa and Asia. Pj plants have flexible branches, a green–brown, a twisted stem, and curving multi-seeded pods with a hardened pericarp and flattened^25^. Pj plants can survive in a very harsh and poor soil environment, control erosion, improve soil fertility, and reduce soil salinity in drylands. Due to invasive properties, Pj plants grow as much as 9 m in length and 50 cm in diameter. The fruit is a non-dehiscent curved pod of 10–15 cm in length, 0.8–1.0 cm in width, 2–4 mm thick, and contains a hard endocarp, soft and heavy mesocarp and woody endocarp with many seeds^24^. The pods generated by the *Prosopis* species are legume pods containing high levels of carbohydrates, protein, and starch used as animal feed for the past decades. *Prosopis* species are made up of exocarps, fibrous endocarps and fleshy mesocarps. The nutritional value of a plant might vary depending on the phenological stage, species, plant portion, world region, and growing climatic conditions^26^.

Pj plant has been investigated for several applications, leaf extract to treat sore eyes, sore throat, diarrhoea, and open wounds^27^ as biopesticides^28^, gums for antioxidants^29^, extracts for antiplasmodial activity^30^, seed powder for the removal of Pb (II)^31^ and all parts of the plant for antibacterial activity^32^. Pj plants have higher carbon and less hydrogen content, leading to the best carbon-effective ratio for biorefinery. Pj pods were used as a raw material in a few fermentations to produce cellulolytic enzymes^33^, xylanase^34^, oxytetracycline^35^, ethanol^36^, bio-oil^37^ and biohydrogen^38^ and biochar production under pyrolysis^39^. To the best of our knowledge, the structural characterization of Pj pods was not yet reported.

The systemic characterization of biomass is essential for biofuel production^40^. The physicochemical properties of biomass help design the biomass conversion process, including feeding, conversion, sorting of intermediate products, and collection of products. Biomass physical, chemical and thermal properties are related to the gasification process, and the knowledge of biomass composition is also essential for solid waste management^41^. Therefore, the present study deals with the proximate, ultimate, biochemical and thermal properties of the *Prosopis juliflora* (Pj) pods to aid the selection of Pj pods as a feedstock for bioenergy and value-added products.

## Materials and methods

### Pj pods collection and size reduction

Pj pods (Common name: algarroba pods) were collected from Pudukkottai, Tamil Nadu, India, as per the ASTM International standards—ASTM E1757-1. The plant biomass was identified by botanist Dr Sam Aldrin, SASTRA Deemed University, India. The raw pods were collected from the same region for the entire study to prevent changes due to different nutritional content. The collected pods were washed completely with tap water and dried under sunlight. The dried pods were ground to achieve a 0.4–0.1 mm particle size using an ASTM sieve^35^.

### Proximate analysis

Proximate analysis of Pj pod powder was conducted to estimate the moisture, volatile matter, ash content and fixed carbon^40^. Essential biofuel quality can be determined by proximate and ultimate analysis^42^.

The conventional oven-dry method (NREL) was used to estimate the moisture content according to ASTM E1756-01. Briefly, 10 g of Pj pods powder was weighed in the crucible and heated in a hot air oven at 105 °C for 4 h. Pj pods powder was removed from the crucible and allowed to cool in a desiccator. The moisture content of Pj pods was calculated using Eq. (1)^40^.

1 $${\text{Moisture}}\left( {{\% }} \right) \, { = 1 - }\left[ {\frac{{\text{a}} - {\text{b}}}{{\text{c}}}} \right]{*100}$$

where a is crucible weight along with Pj pods powder; b is crucible weight; c is the weight of Pj pods powder as received after heating.

The volatile matter was determined according to ASTM standard E-872. Moisture-removed pods powder was taken into a covered crucible, ignified in a muffle furnace at 925 °C for 7 min and allowed to cool in a desiccator. The difference in weight loss was expressed as volatile matter and calculated using Eq. (2)^40^.

2 $${\text{C \% = }}\frac{{\left[ {\left( {\text{a}} - {\text{b}} \right)} \right]}}{{\text{a}}}{*100}$$

where a is the weight of Pj pods powder; b, the weight of Pj pods powder after heating; c, weight loss.

The leftover Pj pods powder from the estimated volatile matter was again ignified in a muffle furnace at 575 °C for 1 h, according to ASTM standard D1102. The difference in weight corresponds to the percentage of ash present in the Pj pods^40^.

Subsequently, the fixed carbon (FC) content was estimated as the difference in weight of moisture, volatile matter and ash content from the initial biomass. The fixed carbon content of Pj pods was calculated using Eq. (3)^40^.

3 $$\% \;{\text{ Fixed }}\;{\text{carbon }}\;({\text{FC}}) \, = { 1}00\; - \;{\text{moisture}}\; \, \left( \% \right)\; \, - \, \;{\text{volatile}}\;{\text{ matter}}\; \, \left( \% \right)\; - \;{\text{ash}}\; \, \left( \% \right)$$

The elemental composition of Pj pod powder, including carbon, nitrogen, oxygen, hydrogen and sulfur, was determined^40^.

### Ultimate analysis

CHNS analysis was carried out using CHNS Elemental analyser model Elemental Vario EL III Germany, analysed at CECRI Karaikkudi, India.

X-ray fluorescence spectrophotometry was used to estimate the mineral composition of the Pj pods. Briefly, the Pj pods powder was mixed with boric acid as a binder under high pressure (22 tons) to make a pellet. The pellet results were recorded in X-ray fluorescence (XRF) spectrometer S8 Tiger, Bruker AXS, Germany, analysed at SASTRA Deemed University, Thanjavur, India, using a 4-kW rhodium anode X-ray tube^43^.

### Biochemical analysis

Pulping and bleaching are two crucial steps to extracting cellulose effectively from lignocellulosic biomass^44^. The acid bleach method was used to isolate and estimate the Pj pods' cellulose. Briefly, 5 g of Pj pods powder was loaded in a Soxhlet apparatus with water and ethanol and reflexed for 8 h to eliminate the wax. The dewaxed Pj pods powder was bleached using 1.5% sodium chlorite for 2 h at 70 °C at pH 3.5. The bleaching procedure was repeated until a consistent white colour was obtained, indicating pure cellulose. The obtained cellulose was filtered, dried and weighed^45^.

The filtrate from the acid bleaching step was treated with 1 M NaOH at 65 °C for 2 h, neutralized with 6 M HCl at pH 5.5 and precipitated using $${\raise0.7ex\hbox{$1$} \!\mathord{\left/ {\vphantom {1 3}}\right.\kern-\nulldelimiterspace} \!\lower0.7ex\hbox{$3$}}$$ volume of ice-cold ethanol. The collected pellet was washed several times with distilled water to remove excess sodium hydroxide and centrifuged to obtain a hemicellulose pellet. The collected hemicellulose was dried in a hot air oven for 24 h and weighed^45^.

Acid hydrolysis was used for the isolation of lignin. Briefly, 1 g of Pj pod powder was hydrolyzed with 72% sulfuric acid, autoclaved for 2 h at 121 °C and filtered off. The filtrate was considered acid-soluble lignin, and the residue was acid-insoluble lignin. Further, the acid-soluble lignin was estimated using a UV–Visible spectrophotometer and Eqs. (4) & (5)^46^.

4 $${\text{Acid}} \;s{\text{oluble}} \;{\text{lignin}} \;\left( \% \right) = \frac{{{\text{UV}} \;{\text{absorbance}}\;*\;{\text{volume}}\;{\text{ of}}\; {\text{filtrate}}\;*\;{\text{Dilution}}}}{{ {\upvarepsilon }\;*\;{\text{ODW}} \;{\text{sample}}*\;{\text{path}} \;{\text{length}}}}*100$$

5 $${\text{Dilution = }}\frac{{{\text{volume }}\;{\text{of}}\;{\text{ sample}}\;{ + }\;{\text{volume }}\;{\text{of}}\;{\text{ diluting}}\;{\text{ solvent}}}}{{{\text{ Volume}}\;{\text{ of }}\;{\text{sample}}}}$$

UV (abs), average absorbance for sample; ε, absorptivity (specific wavelength); ODW sample, sample weight in mg; path length, the path length of UV–Vis cell in cm.

### Determination of extractives

Ethanol and benzene extract fat, wax, oil, gums, sugars and other pigmenting components from LCB. In a Soxhlet apparatus, 2.5 g of dried Pj pods powder was extracted with 150 mL of ethanol at 70 °C and reflexed for 8 h. The extracted Pj pods powder was dried in a hot air oven at 105 °C for 2 h. The weight difference between moisture-free pods and extractive complimentary pods powder is represented as extractives^44^.

### Protein estimation

The Kjeldahl method was adopted to estimate total nitrogen using a kelplus macro block digestion system (Model KES 12 L)^47^. Briefly, 0.5 g of Pj pods powder was mixed with 10 mL of concentrated sulfuric acid and 5 g of catalyst mixture (250 g of potassium Sulphate, 50 g of cupric Sulphate and 5 g of metallic selenium powder in 50:10:1). The mixture was taken in a digestion tube, loaded into the digester, and heated to 400 °C until the mixture turned light green, indicating the complete digestion process. The digested sample was distilled in a kelplus distylemba distillation system with a hose connected (Model classic dx). One side of the hose was connected with a conical flask and loaded with 20 mL of 4% boric acid and mixed indicator (0.066 g of methyl red and 0.099 g of bromocresol green dissolved in 95% of alcohol). The other side of the hose was loaded with 1 M NaOH and distilled. The digested sample was heated by passing the vapour constantly, and the released ammonia was absorbed into a conical flask. With the absorption of ammonia, the pinkish colour turned to green colour. The collected distillate was titrated against 0.02 N of sulfuric acid until the sample colour was changed from green to pink. Micro Kjeldahl total nitrogen was estimated from the titer values. Crude protein was calculated using Eq. (6) from the estimated nitrogen, assuming that nitrogen constitutes 16.5% of protein^48^.

6 $${\text{Crude}}\;{\text{ protein}}\;{\text{ content}}\; \, \left( \% \right) = {\text{ Micro}}\;{\text{ Kjeldahl}}\;{\text{ N}}_{{2}} \left( \% \right)\;*\;{6}.{25}$$

### Starch estimation

The anthrone method was used to estimate the starch. Briefly, the 0.1 g Pj pods powder was mixed with 25 mL 80% of hot ethanol and 5 mL of distilled water. The mixture was stirred thoroughly, centrifuged, and decanted off. The residue was again centrifuged with 30 mL of 80% hot ethanol. Two extracts were combined under pressure in a boiling water bath. The alcoholic extract was mixed with 5 mL of distilled water and 6.5 mL of perchloric acid. The mixture was stirred for 30 min at room temperature and centrifuged. The pellet was suspended in 5 mL of distilled water; the perchloric acid extraction was repeated and centrifuged. Two extracts were combined, diluted to 100 mL and filtered.

Further, 5 mL of the filtrate was diluted to 100 mL with distilled water and used for starch analysis. The starch solution, 1 mL, was diluted with 1 mL of water, and 10 mL of anthrone reagent was added. The mixture was boiled in a boiling water bath for 12 min, cooled, and the amount of glucose was determined by a spectrophotometer. Starch was estimated from the glucose, assuming the 0.9 g starch yields 1 g glucose. Anthrone reagent was prepared as 10 mg of anthrone in 10 mL of concentrated sulfuric acid^49^.

### Thermal analysis

The calorific value of Pj pods powder was determined with a Bomb calorimeter (Toshniwal, India). In a bomb, 0.46 g of moisture-free Pj pods powder was taken in a crucible, kept inside the bomb, and filled with 30 bar pressure of oxygen to combust the Pj pods powder.

TGA was performed in a Thermal analyzer^50^of model SDT Q-600 to identify the thermal stability of Pj pods, cellulose and hemicellulose. The thermal analysis was carried out under a nitrogen atmosphere of 0.5 lb/in^2^ with a 100 mL/min flow rate. The different stages of degradation were observed when the components started heating from 37 to 1000 °C.

### Instrumental analysis

The functional group of Pj pods, cellulose and hemicellulose were determined by Fourier Transform Infra-red Spectroscopy Spectrum one, Perkin Elmer USA^40^ at SASTRA Deemed University. The wavelength was measured in transmission mode from 400 to 4000 cm^−1^ by the ATR (Attenuated Total Reflectance) method^51^. Methanol was used to clean the ATR crystal before use. Later, the crystal was scooped with samples until they covered the surface area of the crystal and pressed with a mechanical anvil.

CP/MAS ^13^C spectrum of cellulose and hemicellulose was studied by Bruker Avance HD 500 MHz spectrophotometer from IITM, SAIF, CSAIF, Chennai, India^44^. The following condition was analyzed: resonance frequency at 125.76 MHZ; proton 90° pulse sequence was 4.30 µs; contact pulse 3300 µs, recycle delay was 5 s; acquisition time 0.02 s; 1024 scan per spectrum were used.

The SEM imaging of dried pods, acid-treated pods, cellulose and hemicellulose, was studied by Vega 2 TE-SCAN microscope with a speeding voltage of 10.0 kV. The magnification of the isolated cellulose was focused on up to 500X^44^.

The isolated cellulose crystallinity index was analysedusingPANalyticalXpert3^50^ at Bishop Heber College, Trichy, India.

X-ray Fluorescence Spectroscopy analysis was conducted to estimate the chemical elements in the Pj pods powder. The mineral composition was studied by Bruker SPECTRA plus for S8 TIGER X-ray Fluorescence Spectroscopy^43^.

## Results and discussion

### Proximate analysis

The proximate and ultimate analyses were used to determine the composition of Pj pods^52^. Table 1 shows the proximate analysis, including Pj pods' volatile matter, ash content, moisture content and fixed carbon. Pj pods powder has less moisture content, 7.89 ± 0.002% w/w and could be a good source for energy conversion. In general, moisture in biomass reduces its heating value^53^ and decreases the yield of biomass combustion. Moisture content is represented as the quantity of water present per unit of Pj pods powder, and the low moisture content yields more combustion. High moisture content will decrease the yield of biomass combustion^54^. Pj pods showed a higher volatile matter content of 87.67 ± 0.002% w/w, which could be a valuable source for pyrolysis. The fuel reactivity has also been influenced by volatile matter^55^. The high volatile content of biomass has a propensity to easily burn and degrade during the thermochemical reaction, ideally yielding a higher proportion of bio-oil during the pyrolysis^56^.

**Table 1:** Proximate analysis of Pj pods on a dry basis.

S. no	Parameter	Pj pods (the present study) % w/w	Reported		
1	Moisture	7.90 ± 0.002	9.48% from *Prosopis juliflora* bark fibres^50^	7.75 (%w) from Sesbania branches^57^	8.58% *Prosopis juliflora* fibres^58^
2	Volatile matter	87.67 ± 0.002	79.7% from mesquite pod bagasse^59^	82.6 (%w) from Sesbania branches^57^	77.5% from *Prosopis juliflora wood*^60^
3	Ash	0.21 ± 0.002	3.20% from mesquite pod bagasse^59^	1.13 (%w)Sesbania wood^57^	1.97% from olive tree pruning residue^61^
4	Fixed carbon	4.23 ± 0.002	9.95 (%w) Sesbania leaf^57^	18.6% from *Lantana camera*^62^	20.78% from cherry stalk^42^

Ash is composed of mineral and inorganic matter of biomass and affects the combustion rate of biomass^40^. The amount of total minerals in biomass indicates the ash content or total ash^63^. The higher and lower heating values, the amount of solid material to release, dust particles in combustion gases, boiler fouling, and corrosion are all strongly correlated with the ash content^64^. In addition, a high amount of ash content acts as a heat sink during pyrolysis or combustion that will reduce the fuel energy process^65^ and biomass with a greater ash content generates more char leftovers during pyrolysis^56^. Pj pods with a low ash content of 0.21 ± 0.002% w/w, comprising phosphorous, silica, aluminium, sodium, iron, calcium, and potassium, could be better feedstock for fuel energy production. However, ash is one example of a solid fraction that has the potential to be used in agriculture because of its high nitrogen, phosphorous, and potassium content^2^.

Fixed carbon (FC) is the amount of leftover Pj pods powder after releasing volatile matter, moisture, and ash^40^. The high amount of fixed carbon represents a high energy level. In most gasifiers, the conversion of FC into gases dictates the pace of gasification and its yield, making fixed carbon a significant parameter for gasification study^66^. Knowing fixed carbon's shape and hardness can aid choose the right combustion equipment since they reveal a fuel's caking qualities^67^. The high volatile matter will increase biochar yield in thermochemical conversion processes. Pj pods have a fixed carbon content of 4.23 ± 0.002% w/w, producing low char during combustion.

### Ultimate analysis

The elemental composition of Pj pods was determined by ultimate analysis (Table 2). The low values of nitrogen and sulfur in Pj pods assure a low rate of nitrogen oxide during biochar production^68^, low greenhouse emissions^42^ and thermochemical conversion processes such as pyrolysis, torrefaction and gasification^2^ and that can produce streams of liquid, solid, and gaseous products. Higher carbon content and a lower oxygen content must result in a higher heating value since the carbon content of biomass is directly correlated with the heating value, and the oxygen content is inversely correlated with the heating value^69^. The amount of carbon and oxygen are directly correlated with the heating value. Pj pods have a high amount of carbon, meaning that the more significant percentage of carbon, the higher the heating value of the fuel.

**Table 2:** Ultimate analysis of Pj pods on a dry basis.

S. no	Contents	The present study (%) w/w	Mesquite pods bagasse^59^	Sesbania leaf (%)
1	Carbon	41.77	43.29	41.03^57^
2	Nitrogen	3.59	4.62	3.72^57^
3	Sulfur	26.30	0.21	0.31^57^
4	Hydrogen	6.55	5.59	5.99^57^
5	Oxygen	21.8	43.09	35.04^57^

Additionally, a more significant oxygen content decreased the heating value of Pj pods, and biomass nitrogen content is crucial for evaluating nitrogen oxide (NOx) emissions, an air pollutant^64^. One of the primary gases emitted during the thermal reactions of biomass is a nitrogen oxide (NOx). Therefore, these emissions need to be controlled as they affect human health. (NOx) consist of seven oxides that are considered the most emissions in an air^70^. When burning fuel, NOx emissions can be reduced in seven major methods by employing a catalytic combustion system, reduced air preheat, low NOx burner to lower the peak temperature, air staging and natural gas burning. Similarly, the ozone-depleting NO_2_ can also be treated in a similar way^70^.

As determined by XRF, the micronutrients Zn, Mn, P, Cu, Ca, Mg, Na, K, and Fe were present in the whole Pj pods (Table 3). The micro and macronutrients present in Pj pods are related to the properties of the soil where PJ pods trees have grown. Non-essential metal such as Lead (Pb) is present at high concentrations in Pj pods (Table 3). The presence of lead will be toxic to the human ecosystem as well as animals. The high number of lead will affect the animal's food chain and cause soil erosion^71^.

**Table 3:** Micro and macronutrients present in Pj pods.

Minerals	Present study (%)	Mesquite pods collected from Guayaquil, Ecuador (%)
K	0.090	0.46^47^
Cl	0.047	–
Pb	0.027	–
Ca	0.016	0.11^47^
Si	0.006	–
S	0.004	–
P	0.004	0.094^47^
Mo	0.003	–
Fe	0.003	0.13^47^
Mg	0.002	0.0097^47^
Na	0.002	0.995^47^

### Biochemical analysis

Chemical analysis data for biomass is crucial information when examining the reaction properties of solid fuels. The term "holocellulose" refers to the total cellulose, which is made up of cellulose and hemicelluloses, and extractives constitute the significant portion of Pj pods powder, 88% with less protein, lignin and starch (Table 4). During pyrolysis, biomass with a higher extractive content yields more liquid products. Acid-soluble and acid-insoluble lignins were isolated and estimated through the acid hydrolysis method. Pj pods contain an almost negligible amount of lignin, 4.71 ± 0.12% w/w. Pj bark has been reported for a good amount of lignin,17.11%^50^. Lignin is the major obstacle to biomass conversion as it inhibits microorganisms' growth^72^.

**Table 4:** Biochemical analysis of Pj pods.

S.no	Contents	The present study (%) w/w	Reported (%)	
1	Cellulose	26.60 ± 0.01	61.65	*Prosopis juliflora* bark^50^
2	Lignin	4.71 ± 0.12	17.11	
3	Hemicellulose	30.86 ± 0.27	16.14	
4	Extractives	30.56 ± 0.008	52.18 ± 0.03	*Prosopis juliflora* bark^60^
5	Proteins	11.63 ± 0.12	10.15	*Prosopis juliflora* pods^47^
6	Starch	1.1 ± 0.06	1.2 ± 0.9	Waxy maise^73^

Sodium chlorite-based bleaching is an effective method to isolate cellulose from biomass. Alkaline treatment for the isolation of hemicellulose was a widely reported method, as it effectively cleaves the ester bond between lignin and hemicellulose^44,45,72^. Pj pods constitute 57.76 ± 0.14% w/w holocellulose less than the Pj bark, 77%^50^.

The amount of protein in Pj pods was estimated by the micro Kjeldahl method. Pj pods have shown a good amount of protein, 11.63 ± 0.12% w/w, an excellent alternative and low-cost animal feed. Further, the exact protein composition has to explore to assess the nutritional value, net protein utilization and digestibility. The protein content of Pj pods from Ecuador was reported as 10.1% (dry basis)^47^ and Pj seeds as 4.5%^54^.

With the high amount of hollocellulose and less lignin content of 4.71 ± 0.12% w/w, Pj pods could be an effective source for energy conversion through biochemical approaches. The negligible amount of starch 1.23 ± 0.06% w/w was determined, similar to starch obtained from waxy maize starch 1.2 ± 0.9%^73^, and the high amount of extractives needs further evaluation.

### Thermal analysis

The calorific value is the heat released per unit of biomass during combustion. The HHV represents the gross calorific value and is influenced by elemental composition, moisture and ash content^74^. The higher HHV also indicates the Pj pods significance as a bioenergy source. The HHV value was calculated by using Eq. (7) obtained by the International Energy Agency (IEA), and the actual values can be seen in Eq. (8)^69^.

7 $$\begin{gathered} {\text{HHV }}\left( {{\text{MJ}}/{\text{kg}}} \right) \, = { 1}.{1783 }\left( {\% {\text{ H}}} \right) \, + \, 0.{3491}\left( {\% {\text{ C}}} \right) - \, 0.0{211}\left( {\% {\text{A}}} \right) \, \hfill \\ \quad \quad \quad \quad \quad \quad \quad \quad - \, 0.0{151}\left( {\% {\text{ N}}} \right) \, - \, 0.{1}0{34}\left( {\% {\text{ O}}_{{2}} } \right) \, + {\text{ hg}}0.{1}00{5 }\left( {\% {\text{ S}}} \right) \hfill \\ \end{gathered}$$

8 $$\begin{gathered} {\text{HHV}}\left( {{\text{MJ}}/{\text{kg}}} \right) \, = { 1}.{1783}*{ 6}.{55 } + 0.{3491}*{ 41}.{77 }{-} \, 0.0{211 }*0.{21 } \hfill \\ \quad \quad \quad \quad \quad \quad \quad \quad {-} \, 0.0{151 }*{ 3}.{59 }{-} \, 0.{1}0{34 }*{ 21}.{8 } + 0.{1}00{5}*{ 26}.{3}0 \hfill \\ \end{gathered}$$

Here, the HHV of Pj pods was calculated using the elemental composition of Pj pods as 22.63 MJ/kg. The energy produced by Pj pods using a bomb calorimeter was 17.073 MJ/kg during combustion. The obtained HHV values from the bomb calorimeter could be compared with other biomass, such as corn cobs^68^ and 17.61 MJ/kg from olive tree pruning residue^61^.

**Figure 1:**
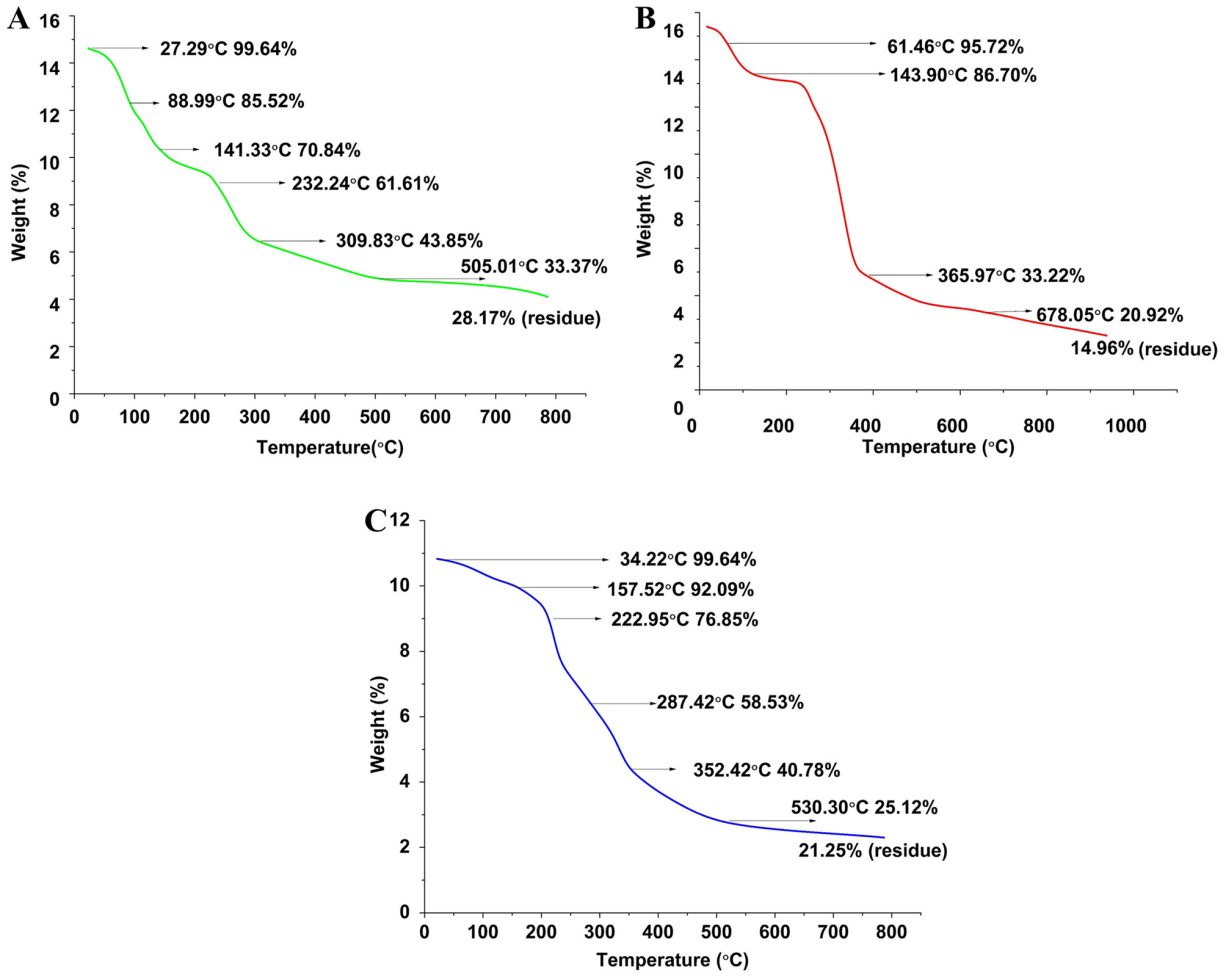
TGA profile of (**A**) Pj pods, (**B**) cellulose (**C**) hemicellulose.

To provide a prior assessment of the pyrolysis behaviour of the biomass being evaluated, thermo-gravimetric analysis TGA was carried out. The thermograms of Pj pods powder, cellulose and hemicellulose are shown in (Fig. 1). All the isolated polymers and Pj pods powder TGA (0–1000 °C) was recorded as weight loss (% w/w). The thermal cleavage of the carbohydrate monomer units causes cellulose and hemicellulose to volatilize when heated in an environment without oxygen in the presence of nitrogen.

Pj pods TGA first stage was recorded at 88.99 °C (Fig. 1A) with 14.125% weight loss due to the evaporation of moisture and volatile compounds. The significant devolatilization of Pj pods occurred between 140 and 350 °C at this point, and a significant weight loss, 41.670%, was reduced and related to the degradation of α cellulose and lignin and hemicellulose^75^. This phenomenon is due to the breakdown of cellulose chains, and the dehydration, decarboxylation, and breakdown of glycosyl cellulose units could all be contributing factors^75^. The final stage of depolymerization of carbon residues was observed between 400 and 500 °C. Additionally, lignin degradation is seen in all the stages, and no further weight loss was recorded after that^75^. 71.83% of weight loss of Pj pods powder was recorded during TGA. A similar thermal degradation pattern was observed in *Cassia fistula* peel and *Syzygium cumin* seeds^76^, *Eragrostisairoides, Imperata cylindrica*l feedstocks, and Coconut husk waste^77^.

In cellulose isolation, the reaction progresses in three stages^17^. The isolated cellulose degradation occurs at 150 °C with an initial weight loss of 9.65% (Fig. 1B). When the initial decomposition stage reaches 200–300 °C, the weight of cellulose begins to decline gradually. This steady decline is typically caused by dehydration in addition to the initial decomposition stage^17^. Most cellulose degradation occurs in the abrupt curve following the initial decomposition stage^17^. The significant cellulose weight loss, 52.85%, occurred between 200 to 380 °C due to depolymerization. But the crystalline structure of cellulose is thermally stable and degraded slowly till 380 °C^17^. Finally, carbon residues were depolymerized completely by 800 ℃. Overall, 85.04% of weight loss of Pj pods cellulose was determined during the tested conditions. The thermal degradation peaks of the isolated cellulose are similar to the literature on cellulose isolated from jackfruit peel^44^, diaper waste^78^, *Calophyllum inum* cake^79^, orange and lychee peel^80^.

The degradation of isolated hemicellulose started at 37 °C (Fig. 1C). Initial moisture and volatile matter evaporation were observed until 157 °C with a weight loss of 7.55%. The weight loss due to depolymerization of hemicellulose, 36.07%, occurred between 200 and 353 °C. Because the hemicellulose decomposes first between 220 and 315 °C due to more thermal liability. But the significant weight loss occurred between 400 and 600, with a total weight loss of 78.75%. A similar pattern of thermal degradation was reported for the hemicellulose isolated from corn stalk^81^, *Neolomarckiacadamba*^82^ and sugarcane^83^.

In contrast, lignin decomposes over a much more extensive temperature range of 190–900 °C and is a significantly more heterogeneous polymer when compared to cellulose or hemicelluloses). Therefore, lignin does not exhibit a straightforward weight loss derivate peak^53^. Furthermore, cellulose and hemicellulose share a significant amount of the thermal breakdown temperature interval with lignin^84^.

### Fourier transform infrared spectroscopy

**Figure 2:**
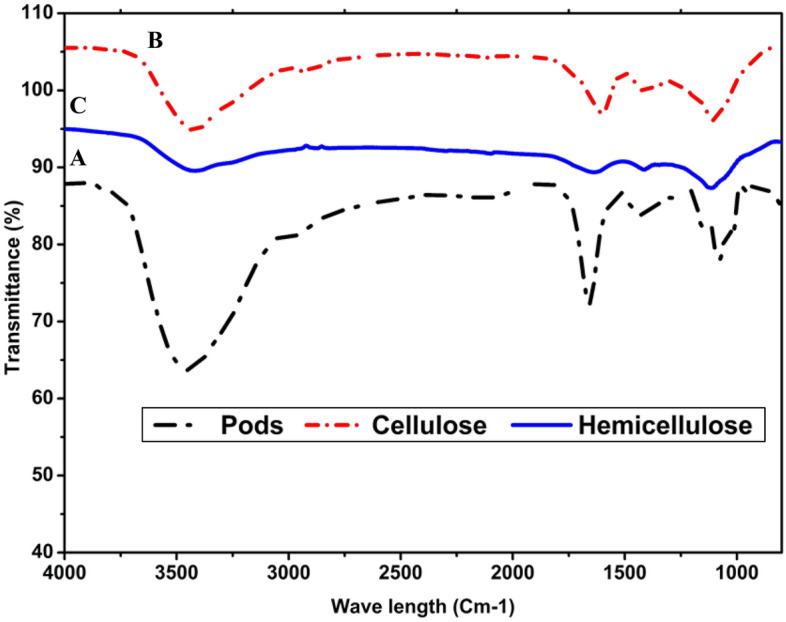
FTIR pattern of (**A**) Pj pods (**B**) cellulose (**C**) hemicellulose.

FTIR spectrum was recorded for Pj pods, cellulose, and hemicellulose to determine the functional groups and assigned bonds (Fig. 2A). In Pj pods, spectral peaks were observed at 3422 cm^−1^ corresponding to the axial angular bending of the O–H bond in hydroxyl groups in α cellulose, showing how the hydroxyl group is broadly distributed throughout the cellulose and hemicellulose structures^85^. Spectral peaks at 1635 cm^−1^ were assigned carbonyl stretching C–O for the acetyl group in hemicellulose and the aldehyde group in lignin. The C–O–C bond deformation of hemicellulose and cellulose was assigned to 1422 cm^−1^. The spectral peak at 1056 cm^−1^ can be assigned to the C–O–C group in Pj pods, and the overlap of stretching vibrations of glycosidic bonds in glucomannan and xylan^85^. A spectral peak of 606 cm^−1^ can be assigned to β glycosidic linkages within sugar units (Fig. 2A). A similar FTIR pattern was observed in *Prosopis juliflora* fibre^50^ and tea leaf brewing waste^86^.

The isolated cellulose FTIR spectrum (Fig. 2B) shows the major spectral peak at 3425 cm^−1^ indicating the fibre axial deformation of OH^40^. The spectral peak at 2927 cm^−1^ can be assigned to C–H symmetric and asymmetric tensile vibration^87^ and deformation of methyl and methylene. The absorption peak of 1603 cm^−1^ presence may be due to water absorption^88^. Peaks at 1408 cm^−1^ were due to CH_2_ bending vibration^89^. The IR peaks at 1106 cm^−1^ are attributed to the C–O asymmetric bridge stretching^44^. The spectral peaks at 617 cm^−1^ indicate -OH out of the plane bending band^90^. A similar pattern of FTIR peaks was observed in isolated cellulose from baby diaper waste^78^, jackfruit peel^44^, *Phyllanthus emblica*^91^, and orange and lychee biorefinery waste^80^.

The isolated hemicellulose FTIR spectrum (Fig. 2C) primarily has a strong band at 3416 cm^-1^ indicating O–H stretching and hydrogen bond. The spectral peak at 2299 cm^−1^ was attributed to the –CH bond deformation of CH_2_ groups. The spectral peak of 1642 cm^-1^ may be due to the absorption of water^88^. The absorption peak at 1113 cm^−1^ was due to ring vibration band C–OH bending^87^, and the stretching vibrations of the side groups COH and glycosidic bonds C–O–C overlapped with the sugar ring vibrations in this region^85^. The absorption peak of 788 cm^−1^ can be assigned to the C1 group frequency in the β -glycosidic linkages between sugar units^87^. The spectral peak of 1412 cm^−^^1^was due to –CH and –OH group bending^87^. Similar hemicellulose FTIR peaks were observed in hemicellulose from bamboo^92^, tea leaf brewing waste^86^ and sugarcane bagasse^83^.

### CP/MAS NMR

**Figure 3:**
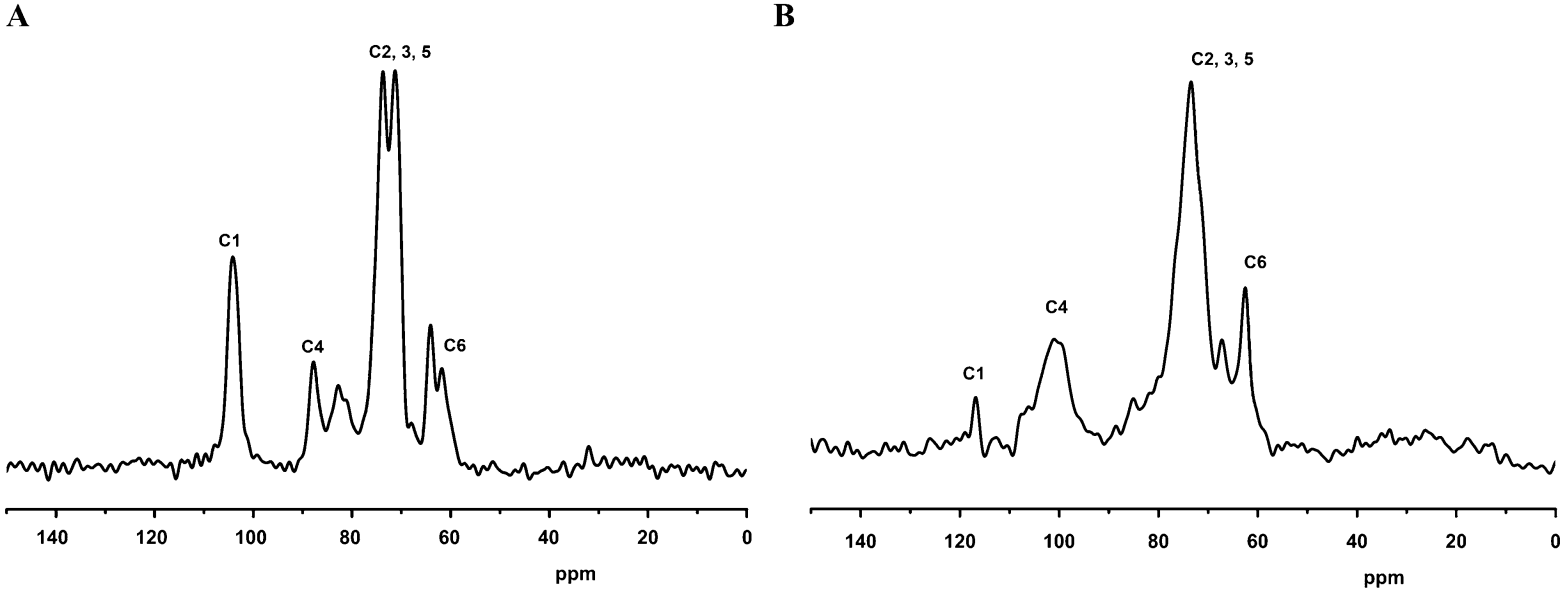
CP/MAS NMR (**A**) cellulose (**B**) hemicellulose.

Cross polarization/magic angle spinning solid-state NMR experiments were performed on CP/MS C13 solid-state NMR for the isolated cellulose (Fig. 3A) and hemicellulose (Fig. 3B). In the cellulose NMR spectrum, C1-C6 carbon peaks were observed in between 60 and 110 ppm which are overlaid on top of signals from less strong lignin and hemicellulose. C1 was assigned at 104.1 ppm, C4 was assigned at 87.90 ppm, 73.42, 72.15 and 71.70 ppm for C2, C3, C5 carbon, respectively. The peaks were divided into two peaks, the lower peak is crystalline, and the higher is the amorphous part of cellulose. C6 carbon was assigned at 61.92 ppm. The presence of crystalline cellulose peaked at 73.42, 72,5 and 71.70 ppm, respectively, and amorphous on the surface of fibres, which peaked at 87.90 and 61.92 ppm, is typically attributed to the splitting of the cellulose C-4 and C-6 resonance lines^93^. The above cellulose peaks are similar to those recorded in isolated cellulose from jackfruit peel^44^, cotton^51^, *Calophylluminophyllum* cake^79^, pomace^91^ and diaper waste^78^.

The C1-C6 carbon peaks of hemicellulose were recorded from 60 to 110 ppm (Fig. 3B). C1 was assigned at 116.91 ppm, C4 was assigned at 101.121 ppm, and 73.42, 73.421 ppm were assigned for C2, C3, C5 carbon, respectively. C6 carbon was assigned at 62.78 ppm. The observed peaks were similar to the hemicellulose of eucalyptus pulp^94^ and Kurrajong wood^95^.

### Scanning electron microscopy

**Figure 4:**
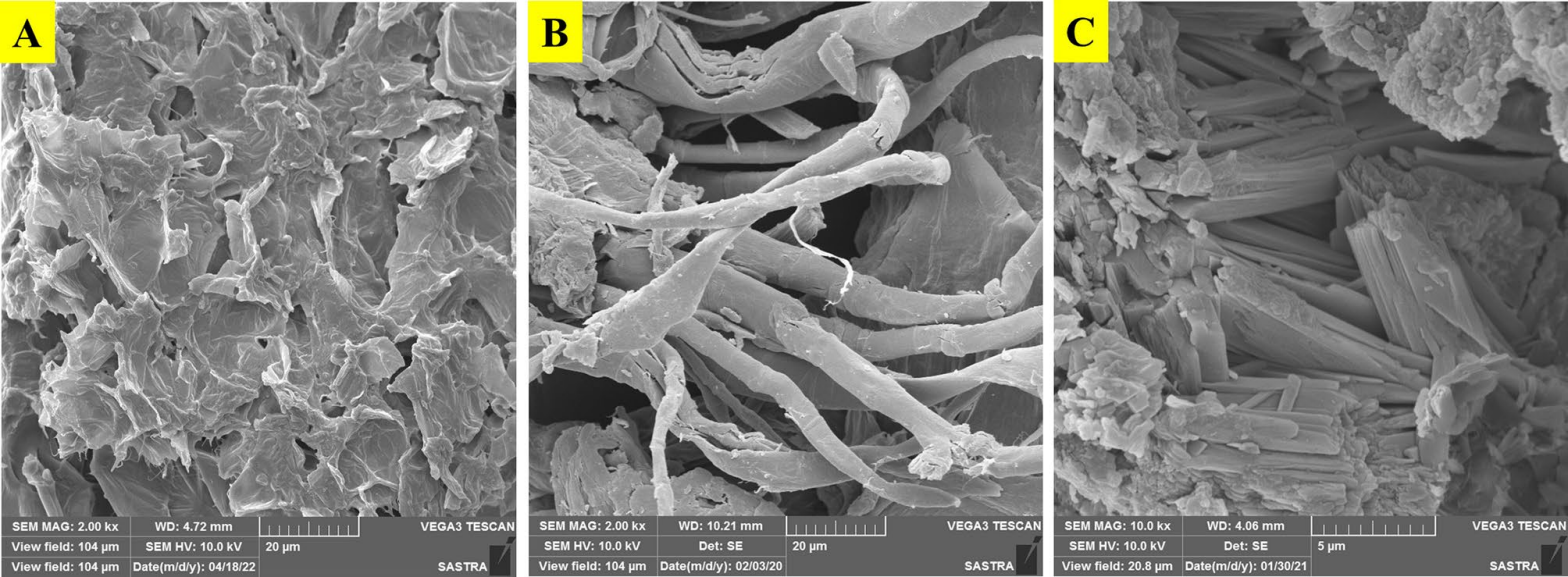
SEM images of (**A**) Pj pods (**B**) cellulose (**C**) hemicellulose.

Pj pods have irregular spherical fibres distributed individually between each other fibres (10 µm), with potentially smaller aspect ratios, giving a large surface area for chemical pretreatment (Fig. 4A). Due to the presence of binding substances like lignin, hemicelluloses, cellulose, the surface of the raw fibre appears uneven. The chemical pretreatments eliminate these binding components, which causes the fibres to come loose from the surface. Whereas the isolated cellulose depicted aggregated irregular bundles with branched tubular microfibril with a length of 20 µm was examined (Fig. 4B). The aggregated fibrils of microcrystalline cellulose composed of hundreds of individual cellulose whisker, which is made up of solid hydrogen bonds^96^. The NaClO_2_ bleaching aids in the removal of remaining lignin and promotes further disintegration, which results in the formation of cellulose microfibrils. The removal of non-cellulosic components is confirmed by the bleached pulp's smoother and more uniform fibril surface^97^. The hydrolysis of nanocrystals is facilitated by the MCC's roughness^96^. During hydrolysis, the amorphous cellulose structure has been removed so that the microfibril structure was formed^98^. Figure 4C shows the hemicellulose is arranged with the vascular bundle and fragmented fibre shape with a clear and rough crystalline surface. SEM images revealed the looped structures support the formulation of various composites.

### XRD analysis for the isolated cellulose

**Figure 5:**
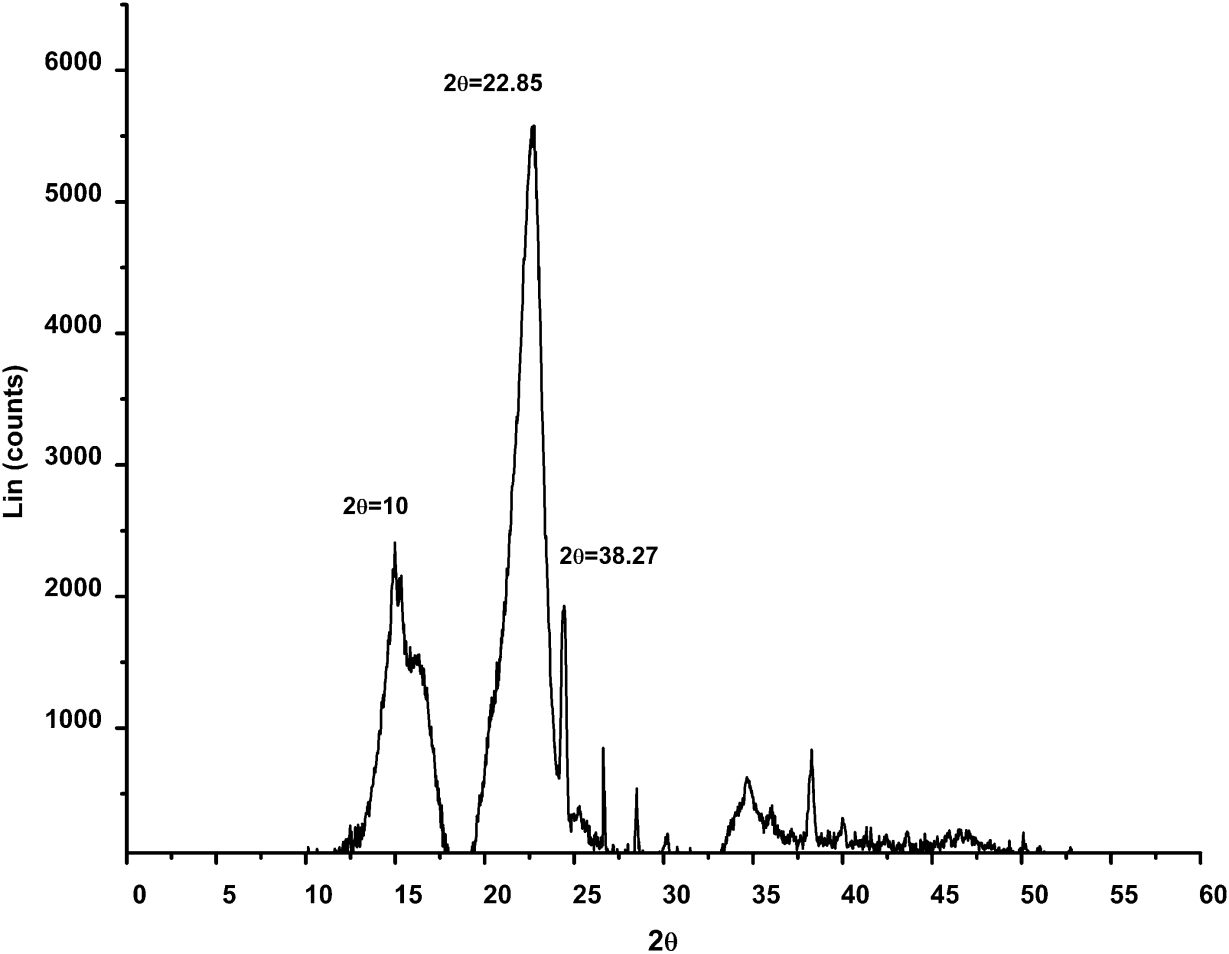
XRD pattern of Pj pods cellulose.

X-ray diffraction was performed to observe the crystalline forms of the isolated cellulose. The cellulose crystallinity will influence the hydrolysis rate of biomass and the source of raw materials. The higher crystallinity is resistant to less reactive and less accessible to hydrolysis. The degree of depolymerization decreases which increases the crystalline index (CrI). There were three typical peaks for Pj pods cellulose at 2θ angles of 2θ = 10º, 22.85º and 38.27º regions related to cellulose type I (crystallographic family of α –cellulose). There is a direct correlation between the degree of crystallinity and the hardness of the samples. The sample becomes more difficult as the crystallinity index rises^75^. Removing amorphous components causes the cellulose index to rise, resulting in the more significant intensity peak associated with bleached fibres^99^. In Pj pods, cellulose, a well-defined crystalline peak, was observed at 2θ = 10, 22.85 and 38.27 (Fig. 5). These peaks are related to the^50^. The Pj pods Crl was found to be 69.43%, which is much higher than the CrI value of sunflower stalk at 51.1% and rice hull 49.8%^100^, *Typha angustata* grass 65.16%^101^ and *Phyllanthus Emblica* 60^91^. The CrI of Pj pods was calculated using Eq. (9) ^51^.

9 $${\text{CI = 100* }}\frac{{{\text{A}}_{{{\text{crystalline}}}} }}{{{\text{A}}_{{{\text{amorphous}}}} {\text{ + A}}_{{{\text{crystalline}}}} }}$$

where A _crystalline_ = area under the crystalline portion of cellulose A_amorphous_ = area under the amorphous portion of the cellulose.

## Conclusion

*Prosopis juliflora* (Pj) pods were characterised using FTIR, SEM, XRD, NMR, HHV and TGA. Lignin, cellulose, and hemicellulose were isolated from Pj pods and evaluated. Pj pods have shown a low high carbon content of 41.77% w/w, a high calorific value of 17.073 MJ/kg, 57.76 ± 0.14% w/w of holocellulose and ash content of 0.21 ± 0.002% w/w. All the parameters were in good agreement with the reported literature. The composition and functional properties of holocellulose, and lignin may differ based on the structure and species of the biomass. However, only marginal differences can be observed in individual biomass based on seasonal and geographical variation. The significant amounts of holocellulose, 57.76 ± 0.14% w/w and the whole carbon content encourage high-value applications of Pj pods in biomass biorefinery. Minor proportions of lignin make it suitable for the fermentative production of biofuels. The present results indicated the significance of the valorization of *Prosopis juliflora* (Pj) pods. However, further in-depth investigations are required to efficiently utilize the same in environmental remediation, biomass-biorefinery and biofuel technologies. Recently our team has reported the hybrid hydrolysis and fermentation of *Prosopis juliflora* (Pj) pods for hydrogen production^38^. The scaleup of hydrogen production is in progress. Further, the combined thermochemical and biochemical routes for refining *Prosopis juliflora* (Pj) pods for bioenergy and other value-added products could contribute to the circular economy.

## Data Availability

The datasets used and/or analyzed during the current study available from the corresponding author on reasonable request.
